# A Case of Placenta Prævia

**Published:** 1866-06

**Authors:** J. W. Brooks

**Affiliations:** Chicago


*A Case of Placenta Prœvia.* By J. W. Brooks, M. D., Chicago.

TO THE EDITORS OF THE CHICAGO MEDICAL JOURNAL.

Allow me briefly to present the notes of a recent case of placenta prævia. At twenty minutes before 2 o'clock P. M., March 26th, 1866, the writer was requested to visit Mrs. McC——n, the friends being greatly alarmed by the amount of her flooding. On my arrival, fifteen minutes before 2 o'clock P. M., they gave me the following account: She commenced flooding a little before 2 A. M., (having suffered from attacks of the same for six weeks previously.) About 7 or 8 o'clock A. M. a physician was called, who made a prescription and left. The prescription being used, and failing to arrest the hemorrhage, I was called at the time before stated, and found her sinking rapidly—restless, pale, pulse feeble, gasping and gaping alternately. On examination, I found the os uteri rigid and undilated; abdomen tense; feeble pain occurring at intervals. Gave one teaspoonful of tinct. camphor in water, plugged the vagina, and sent for my friend, Prof. DeL. Miller, requesting his immediate presence, and ordered camphor water to be freely administered. In forty minutes she had rallied considerably. At the expiration of one hour, a very brisk labor pain expelled a coagulum as large or larger than a pint cup. This was immediately followed by syncope. Examining now, I found the os dilated half an inch, with the placenta attached directly over it. At the same time came another pain with a fearful flow of blood. I restored the plug, grasped the fundus uteri, and pressed my knuckle on the aorta. Dr. M. reaching the house at this time, we found the os dilated nearly one and a half inches, and proceeded forthwith to perforate the placenta and to deliver by podalic version. The flow of blood and liquor amnii was very large.

The placenta followed close upon the child. The mother was well bandaged immediately after the delivery. Nothing unusual has since occurred. The placenta proved to have been perforated in or near the centre.

The child was born in a state of asphyxia. At the expiration, however, of a few minutes it gave a slight gasp, which was soon followed by another. From this the intervals gradually diminished as we used a warm bath and then cold douches, with occasional artificial respiration, till, in one hour and a quarter, we had the pleasure of seeing respirationfully established in a fine healthy boy.

Acting in accordance with the mottoes of Hippocrates—

"Time flies;" "The occasion is fleeting,"—

I believe that the practical lesson to be deduced from this case and its results is, that, in all similar cases, *decision and prompt action are positively necessary* for the safety of mother and child.

